# Validation of the Whooley questions for antenatal depression and anxiety among low-income women in urban South Africa

**DOI:** 10.4102/sajpsychiatry.v23i0.1013

**Published:** 2017-04-11

**Authors:** Carina Marsay, Lenore Manderson, Ugasvaree Subramaney

**Affiliations:** 1 Department of Psychiatry, School of Clinical Medicine, Faculty of Health Sciences, University of the Witwatersrand, South Africa; 2 School of Public Health, University of the Witwatersrand, South Africa; 3 Sterkfontein Hospital, South Africa

## Abstract

**Background/objective:**

In South Africa, approximately 40% of women suffer from depression during pregnancy. Although perinatal depression and anxiety are significant public health problems impacting maternal and infant morbidity and mortality, no routine mental health screening programmes exist in the country. A practical, accurate screening tool is needed to identify cases in these busy, resource-scarce settings.

**Method:**

A convenience sample of 145 women between 22 and 28 weeks gestation was recruited from Rahima Moosa Hospital antenatal clinic in Johannesburg. All women completed a biographical interview, the Edinburgh Postnatal Depression Scale (EPDS), the Whooley questions and a structured clinical interview.

**Results:**

The results demonstrate the sensitivity and specificity of the Whooley questions and the EPDS in identifying depression, anxiety and stress disorders of varying severity. The importance of personal, social and cultural context in influencing the content and expression of these common perinatal conditions was also identified.

**Discussion and conclusion:**

The validity of the Whooley questions in the context of urban South Africa, and the importance of ensuring clinical interviews to supplement any screening tools, is emphasised.

## Introduction

The rate of perinatal depression among South African women living in relative poverty is approximately 40.0%,^1,2,3^ three times the rate documented in high-income countries. Perinatal depression is therefore a significant public health problem in South Africa,^2,4^ with potentially devastating consequences for the mother, foetus, infant and family.^5,6^ Perinatal depression and anxiety are also associated with preterm birth and low birth weight,^7^ malnutrition and poor growth in infants and children,^8,9^ delayed initiation of breastfeeding,^10^ impaired cognition and motor development^11,12,13,14^ and increased child mortality.^15^ Maternal suicide is the leading cause of maternal death in high-income countries with similar estimates to those in low- and middle-income countries.^16,17^ The rate of neonaticide in South Africa is 19.6 per 100 000 live births, with 71.0% of the mothers being identified as the perpetrator.^18^ The high rates of perinatal depression appear to relate to specific risk factors prevalent in South Africa, including poverty,^19^ intimate partner violence,^1,20^ lack of partner support^21,22^ and the high prevalence of HIV in antenatal women (39.0% – 45.0%).^2,23^ Given the high rates and compounding associated risk factors for perinatal depression in South Africa, its early identification and management is important. Screening with referral is a valuable strategy for mitigating the devastating consequences of the illness on mothers and their families. However, more evidence is required to show that screening programmes are effective.^24^

Choosing the best screening tool for a screening programme depends on the context. One needs to consider the population being screened, as well as who will be administering and interpreting the results. As depression screening becomes more routine, the length of the tool also becomes important. Shorter tools, which are less time-consuming, are favoured over longer tools. Case finding questions can identify anxiety and depression with reasonable accuracy.^25^ They are short, do not require scoring or interpretation like pencil and paper tests, and so are more time-effective, and they do not require literacy.

The National Institute of Clinical Excellence (NICE) 2014 Service Guidelines recommend the use of the Whooley case finding questions for screening.^26^ These two questions (see the ‘Whooley questions’ section) address symptoms of depression that are necessary but not sufficient to make a diagnosis of depression. In some settings, a third question is posed, asking the women whether or not they would like help with the problem. There has been much debate about the inclusion of the ‘help’ question in perinatal settings, as it seems to reduce the sensitivity, raising questions of the usefulness of the question.^26,27^ The first two Whooley questions, in contrast, show consistently high sensitivity and moderate specificity in a variety of settings with different populations,^27^ although again with limited evidence to support their use in perinatal settings.^28,29,30^

Gaps exist in the detection of mental illness at the primary care level in South Africa, partly as a result of ineffective screening tools.^31^ Many screening tools have been tested in the country, but most of them appear to be too time-consuming to be administered successfully in busy under-resourced antenatal and postnatal clinics.^32,33^ A study conducted in Cape Town illustrated that it is feasible and acceptable to incorporate mental health screening and depression assessment, with referral, into antenatal clinics,^34,35^ and as a result, the researchers who conducted this have advocated for the use of an accurate, practical three-item screening tool, based on the Whooley case finding questions.^32^

The aim of the study reported in this article was to evaluate the Whooley case finding questions as a potential screening tool, against a clinical interview and the Edinburgh Postnatal Depression Scale (EPDS). The study was conducted in a state hospital in Johannesburg. We determined whether the Whooley questions can be used as a practical, accurate screening tool and whether the addition of the ‘help’ question enhanced its utility.

## Method

### Sample size

A sample size of 145 patients was required to estimate sensitivity and specificity at 75% with 11% precision (rather than 10%), which is reasonable, given the exploratory nature of the study, with a 95% confidence interval, and the prevalence of the diagnosis of 40%.

### Study design

The study was conducted at Rahima Moosa Hospital in Johannesburg, South Africa. Rahima Moosa is a tertiary level mother and child hospital, and a training hospital affiliated with the University of the Witwatersrand. Women who attend the antenatal clinic in this setting all have high-risk pregnancies, defined as a condition that puts the mother and developing foetus at higher-than-normal-risk for complications during birth and pregnancy.^23^ Despite the vulnerability of these women, there is no maternal mental health service provided at Rahima Moosa, and the hospital does not offer a specific adult psychiatric service. In non-emergency cases, women are referred to their nearest mental health community clinic. In the case of an adult psychiatric emergency, women are seen by the child psychiatrist on call and are then referred to the nearby Helen Joseph Hospital, where there is an adult psychiatric unit, for further management.

A convenience sample of women attending the antenatal clinic at Rahima Moosa Hospital was used as only the principal investigator was collecting data, and the volume of women seen daily was high, making consecutive sampling difficult. The inclusion criteria were women able to communicate in English attending the antenatal clinic, 18 years or older, between 22 and 28 weeks pregnant, willing to participate and who provided informed consent forms.

Of a total of 149 women approached to partake, four were not able to communicate adequately in English and thus their interviews were terminated. The remaining 145 patients provided informed consent and participated in the interview. Data were collected between July 2015 and April 2016.

### Assessments

The Whooley questions and the EPDS are screening tools used to detect possible perinatal depression.

#### Edinburgh Postnatal Depression Scale

The most widely recognised screening instrument for perinatal depression is the EPDS. This scale was validated in South Africa, with a group of postnatal women at Rahima Moosa Hospital in 1998.^36^ The EPDS is a 10-item self-report scale that explores symptoms of anxiety and depression experienced in the past 7 days.^37^ It is easy to score with final scores between 0 and 30. The original validation study recommended a cut-off of 10 for possible depression and ≥ 13 for probable depression or psychological distress.^37^ In South Africa, a score of ≥ 13 has been shown to have a specificity of > 76% for both major and minor depression.^36^ In a validation study conducted by Lawrie and colleagues, women were assisted to complete the scale verbally and this proved to be a valid way of administering the screening tool.^36^ In this present study, women were assisted by the principal investigator and gave verbal answers. A score of ≥ 13 was used as a cut-off for probable depression and referral. The anxiety subtest of the EPDS (questions 3–5) was also analysed to determine the effectiveness of the instrument in screening for anxiety disorders in this setting.

#### Whooley questions

The Whooley questions address core symptoms of depression (low mood and lack of interest):

1. During the past month, have you often been bothered by feeling down, depressed or hopeless?
2. During the past month, have you often been bothered by having little interest or pleasure in doing things?
3. A positive test is a ‘yes’ answer to either of those questions, and then a third question is posed:
4. Do you think it is something you want help with?

This third question provides an opportunity for the patient to request help with these symptoms.^29^

#### Clinical interview

The clinical interviews were undertaken by the principal investigator, a psychiatrist, using the NetSCID, an electronic research version, non-patient edition of the Structure Interview of DSM (SCID) as a guide and aligning the diagnoses with the DSM-5 classification. Only the mood and anxiety disorder modules, including stress/trauma related disorders, were administered. The DSM-5 categories of unspecified anxiety and depression were used to categorise subsyndromal but clinically significant symptoms of anxiety and depression, respectively. This is important because subsyndromal symptoms of anxiety and depression can cause similar levels of distress as reported in women with a clear diagnosis^38^ and because perinatal depression and anxiety occur on a continuum of severity.^39^

## Ethical consideration

Ethics clearance was granted by the Human Research Ethics Committee of the University of the Witwatersrand.

## Analysis

Categorical variables were summarised by frequency and percentage tabulation. Continuous variables were described by the mean, standard deviation (SD), median and interquartile range (IQR). For the comparison between demographic and risk factor and diagnosis, patients were classified as having no diagnosis, a diagnosis of depression or a diagnosis of trauma-related/anxiety disorder. The χ^2^ test was used to assess the relationship between categorical risk factors and diagnosis, as well as between the ‘help wanted’ indicator and diagnosis. Fisher’s exact test was used for 2 × 2 tables or where the requirements for the χ^2^ test could not be met. The strength of the associations was measured by Cramer’s V and the phi coefficient, respectively. The following scale of interpretation was used: ≥ 0.50 high association, 0.3–0.49 moderate association, 0.10–0.29 weak association and ≤ 0.10 little or no association. One-way analysis of variance (ANOVA) was used to assess the relationship between age and diagnosis, and the strength of the association was measured by Cohen’s *d*.

The sensitivity and specificity (together with 95% confidence intervals) of the Whooley test, EPDS and EPDS anxiety subscale in identifying the various diagnoses were calculated, with the diagnostic interview used as the reference standard. Here, the diagnoses were considered as follows: (1) any diagnosis versus no diagnosis, (2) anxiety/trauma versus no/any other diagnosis and (3) depressive disorders versus no/any other diagnosis. Trauma-related disorders were grouped with anxiety disorders as there were very few to analyse separately. By considering different cut points for the scales, receiver-operating characteristic (ROC) curves were generated. The impact of the Whooley questions, including the ‘help’ question, on the diagnosis of depression was determined by log-binomial regression. Data analysis was carried out using SAS Version 9.4 for Windows. The 5% significance level was used.

## Results

The mean age of the sample was 31.1 years (range 18–42 years; s.d. = 6.0 year). The majority of women were married/cohabiting (77.9%). Sixty-three (43.5%) women had completed school and 36 (24.8%) had some form of tertiary education, while four (2.8%) women had only attended primary school. The majority of women were working (52.4%), either full-time (39.3%) or part-time (13.1%), 25.0% were unemployed and looking for work, consistent with the general unemployment rate in South Africa,^23^ while 22.6% were unemployed but not looking for work. The median household monthly income was R7000 (IQR R4000 – R12 000; range R1000 – R55 000). Congruent with the urban setting of the study, access to services was above the national average,^40^ 95.9% of the participants had electricity connected to their homes and 82.8% had an inside toilet, while 16.6% had an outside toilet. Only one participant had to use a shared outside toilet. The majority of participants reported that their partners were either very supportive or supportive (86.9%), while a few (13.1%) reported their partners as unsupportive.

Participants were fairly evenly spread between 22 and 28 weeks of pregnancy. The antenatal clinic sees women who have high-risk pregnancies, and in this sample, 95.2% of participants were defined as high risk. It is not unexpected then that 41.4% of these women had experienced a previous miscarriage or stillbirth. Most women (91.0%) in the study group reported that they were happy about the pregnancy, illustrating that mostly the babies were wanted, even if they were unexpected pregnancies. Approximately 19.0% of the participants were HIV-positive; the rest were HIV-negative. This is in contrast to much higher rates of HIV infection recorded in women in antenatal clinics in the rest of the country.^23^

Overall, on the basis of the clinical interview, 56 (38.6%) of the participants were found to have at least one diagnosis of a perinatal mental disorder. Twenty-eight (19.3%) had depression, of whom 16 (11.0%) had major depression. Thirty-two women had diagnosis of either an anxiety disorder (21; 14.5%) or a trauma-related disorder (11; 7.6%). Only 20.0% of women felt that they needed help according to the ‘help’ questions of the Whooley questions, thus having a positive screen. Twenty-seven (18.6%) women had a diagnosis of a past mental illness on the clinical interview, although only 19 (13.1%) received treatment in the past, while eight (30.0%) of these women were untreated for their previous mental illness.

The mean age of those with trauma/anxiety (29.0 years; s.d. = 6.5 y) was significantly lower than that for those without any diagnosis (32.1 years; s.d. = 5.5 y; *p* = 0.040). The effect size was moderate (Cohen’s *d* = 0.55). There were no other significant associations between any other social and demographic variables and having a diagnosis. There was a marginal, weak, association between happiness about pregnancy and diagnosis of depression (Fisher’s exact test; *p* = 0.050; phi coefficient = 0.21); 16.7% of those who are happy about the pregnancy have depression, compared with 46.2% of those who are not happy about the pregnancy. There was also a significant, moderate, association between partner support and diagnosis of depression (Fisher’s exact test; *p* = 0.0072; phi coefficient = 0.34), 12.7% of those who have very supportive partners have depression compared with 52.6% of those who do not have supportive partners. Women diagnosed with depression had a higher proportion of unsupportive partners compared with those not diagnosed with depression (Table 1).

**TABLE 1:** Association between a diagnosis of depression and feeling happy about pregnancy and partner support.

Question		Diagnosis						*p*-value for between-group test	Prevalence ratio (95% confidence interval)	
	
		No mental illness		Depression		Other mental illness				
			
		*n* (89)	Row %	*n* (28)	Row %	*n* (28)	Row %		Depression versus no mental illness	Other mental illness vs. no mental illness
Happy about pregnancy	No	6	46.2	6	46.2	1	7.7	0.050	2.39 (1.22–4.70)	1.14 (0.82–1.57)
	Yes	83	62.9	22	16.7	27	20.5		1	1
Partner support	Not supportive	8	42.1	10	52.6	1	5.3	0.0072	3.44 (1.71–6.95)	1.18 (0.90–1.54)
	Supportive	29	61.7	8	17.0	10	21.3		1.34 (0.58–3.09)	0.99 (0.78–1.24)
	Very supportive	52	65.8	10	12.7	17	215		1	1

Note that the depression group includes four patients with co-morbid trauma/anxiety.

### Whooley questions

**FIGURE 1:**
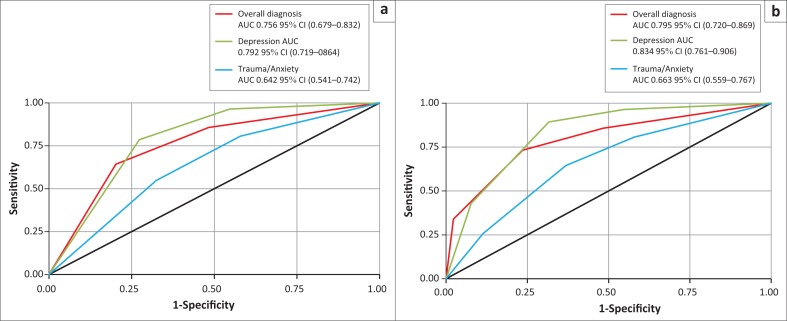
Comparison of ROC curves: ROC curve for the Whooley questions, (a) excluding the ‘help’ question and (b) including the ‘help’ question.

To establish a diagnosis (a positive screen) using the Whooley questions excluding the ‘help’ question, the optimal cut-off point is a score of 2, that is, answering ‘yes’ to the first two questions, giving a sensitivity and specificity of 64.3% and 79.8%, respectively. To establish a diagnosis (a positive screen) using the Whooley questions including the ‘help’ question, the optimal cut-off point is a score of ≥ 2, that is, answering ‘yes’ to two or more of the questions, including the ‘help’ question, giving a sensitivity and specificity of 73.2% and 76.4%, respectively. The sensitivity increased when the ‘help’ question was added (*p* = 0.31) (Figure 1a). The Whooley questions, including the ‘help’ question, had greater discrimination for depression than for anxiety/trauma disorders (Figure 1b).

The specificity of the Whooley questions, including the ‘help’ question (63.2%), was significantly higher than that of the Whooley questions, excluding the ‘help’ question (42.1%) for a diagnosis of trauma/anxiety (*p* = 0.0014). There was a significant association between the ‘help’ question and diagnosis (chi-squared test; *p* < 0.0001; Cramer’s V = 0.48). Those who indicated that they wanted help were more likely to have a diagnosis of depression (51.7%) or anxiety/trauma (31.0%) compared with those who indicated that they did not want help (11.2% and 16.4%, respectively). The impact of the Whooley questions, including the ‘help’ question, on the diagnosis of depression as determined by log-binomial regression is shown in Table 2. The first Whooley question and the ‘help’ question were both significant. The prevalence ratios indicate that ‘yes’ answers for each of these questions are associated with an increased likelihood of a diagnosis of depression. The second Whooley question was not significant. This shows that the ‘help’ question is useful in screening for depression.

**TABLE 2:** Impact of the Whooley questions, including the ‘help’ question, on the diagnosis of depression.

Yes versus no	*p*	Prevalence ratio	95% CI for prevalence ratio	
First Whooley question: yes versus no	0.027	9.99	1.30	76.99
Second Whooley question: yes versus no	0.14	1.86	0.81	4.23
‘Help’ question: yes versus no	0.010	2.22	2.22	4.09

### Edinburgh Postnatal Depression Scale

The median EPDS score was 10 (IQR 6–14; range 0–25). To establish a diagnosis, the optimal cut-off point is a score of ≥ 12, giving a sensitivity and specificity of 78.6% and 84.3% respectively. Although the optimal cut-off points differed (≥ 14 for depression and ≥ 11 for trauma/anxiety), the EPDS seemed to have greater discrimination towards depression than towards anxiety/trauma. There was no significant difference between the Whooley questions, excluding or including the ‘help’ question, and the EPDS when it came to sensitivity for depression. The EPDS had significantly higher specificity for depression (88.0%) than the Whooley questions, whether excluding or including the ‘help’ question (72.6 and 68.4, respectively; Table 3a and Table 3b). The EPDS had slightly lower specificity (60.5%) for anxiety/trauma than the Whooley questions, excluding the ‘help’ question (42.1%).

**TABLE 3a:** Comparison of screening tools, sensitivity and specificity, with confidence intervals.

Variable	Overall diagnosis		Depression		Trauma/anxiety	
		
	Sensitivity (95% CI)	Specificity (95% CI)	Sensitivity (95% CI)	Specificity (95% CI)	Sensitivity (95% CI)	Specificity (95% CI)
Whooley excluding ‘help’ question	64.3% (50.4–76.6)	79.8% (69.9–88.6)	78.6% (59.1–91.7)	72.6% (63.6–80.5)	80.6% (62.5–92.6)	42.1% (32.9–51.7)
Whooley including ‘help’ question	73.2% (59.7–84.2)	76.4% (66.2–84.8)	89.3% (71.8–97.7)	68.4% (59.1–76.7)	64.5% (45.4–80.8)	63.2% (53.6–72.0)
*p*-value for comparison to Whooley (excluding ‘help’)	0.31	0.58	0.28	0.48	0.16	0.0014
EPDS	78.6% (65.6–88.4)	84.3% (75.0–91.1)	89.3% (71.8–97.7)	88.0% (80.7–93.3)	80.6% (62.5–92.6)	60.5% (50.9–69.6)
*p*-value for comparison to Whooley (excluding ‘help’)	0.094	0.43	0.28	0.0031	> 0.99	0.0054
*p*-value for comparison to Whooley (including ‘help’)	0.50	0.18	> 0.99	0.0003	0.16	0.67

EPDS, Edinburgh Postnatal Depression Scale.

**TABLE 3b:** Comparison of screening tools, sensitivity and specificity, with confidence intervals.

Variable	Trauma/anxiety		Trauma		Anxiety	
		
	Sensitivity (95% CI)	Specificity (95% CI)	Sensitivity (95% CI)	Specificity (95% CI)	Sensitivity (95% CI)	Specificity (95% CI)
EPDS anxiety subscale	54.8% (36.0–72.7)	81.6% (73.2–88.2)	63.6% (30.8–89.1)	76.9% (68.8–83.7)	71.4% (47.8–88.7)	62.1% (53.0–71.7)
*p*-value for comparison to Whooley (excluding ‘help’)	0.030	< 0.0001	-	-	-	-
*p*-value for comparison to Whooley (including ‘help’)	0.44	0.0019	-	-	-	-
*p*-value for comparison to EPDS scale	0.030	0.0004	-	-	-	-

EPDS, Edinburgh Postnatal Depression Scale.

### Edinburgh Postnatal Depression Scale anxiety subscale

The median EPDS anxiety subscale score was 5 (IQR 3–7; range 0–9). For the anxiety/trauma diagnosis, the optimal cut-off point is a score of ≥ 7, giving a sensitivity and specificity of 54.8% and 81.6%, respectively. The EPDS anxiety subscale had significantly lower sensitivity (54.8%) for anxiety/trauma than the Whooley questions, excluding the ‘help’ question, (80.6%) and EPDS (80.6%), but significantly higher specificity (81.6%) for anxiety/trauma, than the Whooley questions, excluding the ‘help’ question (42.1%) and EPDS (60.5%).

## Discussion

Screening for anxiety is as important as screening for depression. Despite this, there are few studies on perinatal anxiety and no adequately validated screening tool for anxiety in South Africa. In this study, the rate of perinatal mental disorder was 38.6%. Of those women with disorders, 19.0% had a depressive disorder and 21.4% had trauma/anxiety disorder. This is comparable with other studies that report that anxiety disorders are more prevalent than depressive disorders in women antenatally.^32,41^ Of the patients, 19.0% had a depressive disorder, which is much less than that reported in other South African studies.^1,2,3^ This may be because of the setting and the patient profile. The women in the study overall had good partner support, were not living below the poverty line, had good access to services (indoor plumbing and electricity), were mostly educated, employed and happy about being pregnant. While 13.0% had received a mental health intervention previously, 18.0% were diagnosed with a past history of mental illness. Therefore, some women suffered mental illness without receiving treatment. This is possibly because of a general lack of awareness of mental health within the community, and lack of accessible mental health services. The rate of HIV in this setting is 18.6%, which is lower than other antenatal samples.^23^

A weak association was noted between younger age and a diagnosis. This is in keeping with other literature that describes younger age as a risk factor. There was also a weak association between being happy about the pregnancy, partner support and depression, again in keeping with other South African studies.^1,2,42^

In the original validation study of the Whooley questions, the sensitivity and specificity were 96.0% and 57.0%, respectively, making it a promising screening tool.^43^ There has been much debate about the inclusion of the ‘help’ question as it seems to reduce the sensitivity when asked in a perinatal setting.^44,45^ In this study, the ‘help’ question was valuable because it increased the sensitivity of the Whooley questions from 64.3% to 73.2%. However, because of the relatively small sample size, this difference is not statistically significant. This is a limitation of the study. The other notable difference is that in this study, the Whooley questions did not perform as well in terms of sensitivity as similar studies in other settings, but had higher specificity.^45,46^ This may be because the language of the Whooley questions is complex and more difficult to digest if English is not the patient’s first or primary language. However with a sensitivity and specificity of 73.2% and 76.4%, respectively, when adding the ‘help question’, it still has good utility as a screening tool. The ‘help’ question was also significantly associated with a diagnosis of depression, again suggesting that in this setting the ‘help’ question is valuable. Overall, the EPDS had higher sensitivity and specificity when using a cut-off of ≥ 12 than the Whooley questions, but again these differences are not significant. This makes the Whooley questions, whether including or excluding the ‘help’ question, comparable with the EPDS when screening for antenatal anxiety and depression.

The EPDS anxiety subscale performed poorly as a screening tool for anxiety/trauma disorders with a sensitivity of 54.8%; however, it showed a good specificity of 81.6%. The Whooley questions, excluding the ‘help’ question, show greater sensitivity for the anxiety/trauma diagnosis than the EPDS anxiety subscale. For this reason, the Whooley questions could be used to screen for all perinatal mental disorders including anxiety disorders.

## Conclusion

There is currently no policy on routine screening for perinatal depression and anxiety in South Africa.^35^ The Whooley questions have shown promise as a screening tool in this urban, low-income setting and possibly in other settings. When using the cut-off of ≥ 2, with the inclusion of the ‘help’ questions they show good sensitivity and specificity to depression, anxiety and trauma-related disorders. The questions allow for the early identification of probable antenatal depression and anxiety in about 30% of women attending antenatal clinic. This early identification, if followed by clinical assessment and adequate treatment, will help protect against adverse effects of perinatal depression and anxiety in a significant number of women. The sensitivity and specificity of this tool could be enhanced by either rewording it into more easily understandable language or by translating it into local languages.
